# Spreading depolarization in neurocritical care: a review of SD’S pathophysiological continuum and clinical translation

**DOI:** 10.3389/fnhum.2026.1759247

**Published:** 2026-04-08

**Authors:** Peng Zhang, Xianyun Liu, Jingcun Huang, Yu Tong, Xi Peng

**Affiliations:** 1 The Second Affiliated Hospital of Chongqing Medical University, Chongqing, China; 2 Shenzhen University, Guangdong, China

**Keywords:** delayed cerebral ischemia, electrocorticographic suppression, ketamine neuroprotection, multimodal neuromonitoring, neurocritical care, spreading depolarization

## Abstract

Patients in neurocritical care are often vulnerable to secondary brain injury, wherein spreading depolarization (SD) is identified as a pivotal electrophysiological catalyst. This study consolidates current advancements in SD research, focusing on its clinical translation into neurointensive care. A primary emphasis is the revised and accurate terminology, differentiating the depolarization event (SD) from the concomitant electrophysiological suppression termed cortical spreading depression (CSD), a distinction crucial for interpreting both fundamental and clinical data. Notable advancements in non-invasive detection, especially using full-band scalp electroencephalography (EEG) enhanced by machine learning, currently attain sensitivities of over 85%, with the potential to broaden SD monitoring outside specialized facilities. The integration of SD data with multimodal parameters—such as cerebral microdialysis (which reveals distinctive glutamate surges and metabolic crises) and tissue oxygenation—provides a more comprehension understanding of the underlying pathophysiology. The review highlights dose-dependent treatment strategies, indicating that preliminary clinical studies suggest ketamine’s effectiveness in suppressing SD may rely on maintenance dosages beyond approximately 1.15 mg/kg/h. These converging research fronts establish SD not merely as an epiphenomenon but as a dynamic, actionable biomarker for real-time precision management in brain injury.

## 1 Introduction

Neurocritical illnesses—including traumatic brain injury (TBI), aneurysmal subarachnoid hemorrhage (aSAH), and malignant hemispheric stroke (MHS)—are linked to a significant risk of secondary neuronal injury and unfavorable neurological outcomes (Maas et al., 2017; Lawton and Vates, 2017). Although the severity of original injuries is frequently established in advance, downstream injury cascades, such as neuroinflammation, excitotoxicity, and metabolic crises, represent modifiable treatment targets. Accumulating evidence indicates that SD—waves of nearly complete neuronal and glial depolarization that propagate across gray matter—constitutes a crucial electrophysiological mechanism underpinning secondary damage (Dreier, 2011; Lauritzen et al., 2011).

Spreading depolarization was initially characterized by Leão (1947) in the cortex of rabbits. The contemporary consensus, as delineated by the COSBID research group, explicitly defines SD as the mass depolarization of neurons and glia, while cortical spreading depression (CSD) refers to the suppression of spontaneous electrocorticographic activity that often accompanies SD in the functional cortex (Dreier et al., 2017). This terminological precision is essential for interpreting both basic science and clinical outcomes.

In ischemic stroke, SD is frequently observed as peri-infarct depolarizations (PIDs), which serve as a principal mechanism driving infarct expansion into penumbral areas. In hemorrhagic stroke subtypes like aneurysmal subarachnoid hemorrhage (aSAH), secondary injury significantly influences both early brain injury and delayed cerebral ischemia (DCI), the latter being a major contributor to stroke-related morbidity and mortality. Thus, a thorough analysis of SD dynamics is fundamental for developing effective neuroprotective strategies in stroke care.

The clinical significance of SD is underscored by its high prevalence in acute brain injuries: approaching 100% in MHS (Woitzik et al., 2013), exceeding 60% in aSAH (Fabricius et al., 2008), and approximately 50% in severe TBI (Hartings et al., 2011a). Importantly, specific features of SD, such as clustering behavior and prolonged duration, are strongly correlated with lesion progression and worse functional outcomes (Hartings et al., 2020; Hartings et al., 2011b). The primary objective of this review is to synthesize recent (prioritizing evidence from 2020 onward) advancements in SD pathophysiology, monitoring technologies, and clinical correlations, with a specific emphasis on translation this knowledge into actionable insights for the neurointensivist. While excellent reviews address SD’s basic mechanisms, this synthesis provides a focused update on its evolving role in clinical monitoring and precision therapy. We distinctly highlight several crucial transitions: (1) the essential adoption of standardized COSBID terminology for clinical translation; (2) the emerging feasibility of non-invasive EEG for SD detection, potentially enhancing patient access; (3) the necessity for multimodal integration to interpret SD’s metabolic and hemodynamic context; and (4) a nuanced examination of therapeutic strategies, moving from proof-of-concept toward dose-responsive protocols. By contextualizing these advancements across conditions like TBI, aSAH, and MHS, this review aims to provide a practical framework for SD—guided neurocritical care.

## 2 Pathophysiological continuum of SD

### 2.1 Commencement and advancement

**Figure 1:**
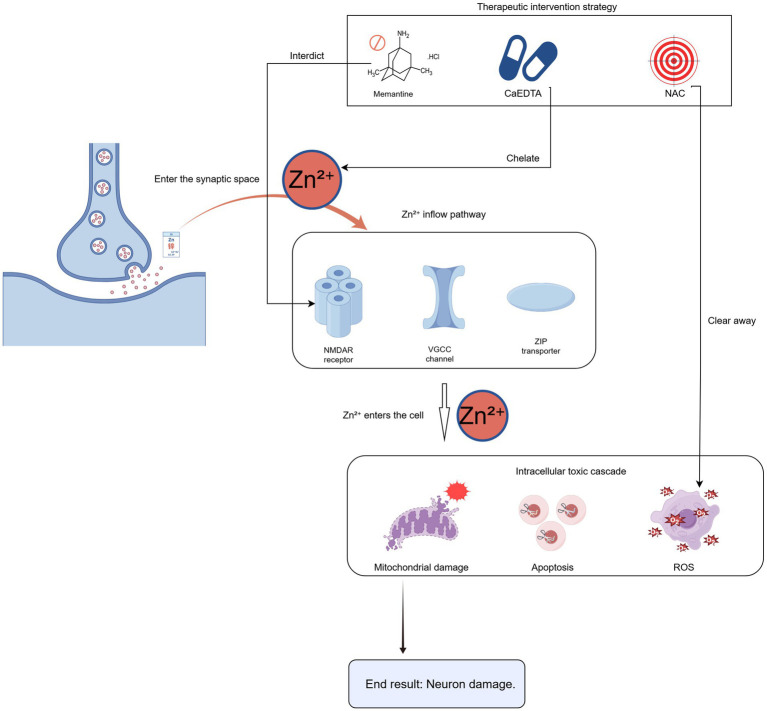
Neurotoxic mechanisms mediated by Zn^2+^ in spreading depolarization.

SD is defined by a near-total breakdown of transmembrane ion gradients. The process is initiated when a threshold of neuronal excitement is exceeded, frequently in metabolically compromised tissue. This triggers a catastrophic transition from a dual Gibbs-Donnan equilibrium to a singular state, releasing over 90% of the Gibbs free energy within seconds and propelling the depolarization wave (Dreier et al., 2013; Lemale et al., 2022). Principal mechanisms include:

1. K^+^-glutamate synergy: When extracellular K^+^ concentration reaches or exceeds 15 mM, it synergizes with glutamate to activate NMDA receptors, facilitating the propagation of SD (Zhou et al., 2013).
2. Zinc-mediated toxicity: Zn^2+^ is co-released with glutamate from synaptic vesicles and enters neurons via multiple pathways (e.g., NMDARs, voltage-gated channels, ZIP transporters). Intracellular Zn^2+^ accumulation disrupts mitochondrial function, exacerbates oxidative stress, and contributes to neuronal death (Figure 1; Carter et al., 2011; Bennett et al., 2024).
3. Neurovascular Coupling Failure: Under pathological conditions, SD can trigger profound vasoconstriction instead of the nomal hyperemic response, resulting in spreading ischemia—a key mechanism for lesion expansion (Dreier et al., 2009; Østergaard et al., 2015; Hinzman et al., 2014).
4. Cerebrovascular Dysregulation: Throughout stroke progression, the structural and functional integrity of the neurovascular unit is significantly disturbed. SD aggravates microcirculatory dysfunction by inducing inverse neurovascular coupling, where pathological vasoconstriction replaces normal vasodilation. This process is especially detrimental in the ischemic penumbra, where tissue survival depends on collateral perfusion. Hemoglobin breakdown products following hemorrhage markedly inhibit nitric oxide bioactivity, creating a self-perpetuating cycle of depolarization-ischemia (Wevers et al., 2021; Ginsberg, 2016; Pensato et al., 2025).

**Figure 2:**
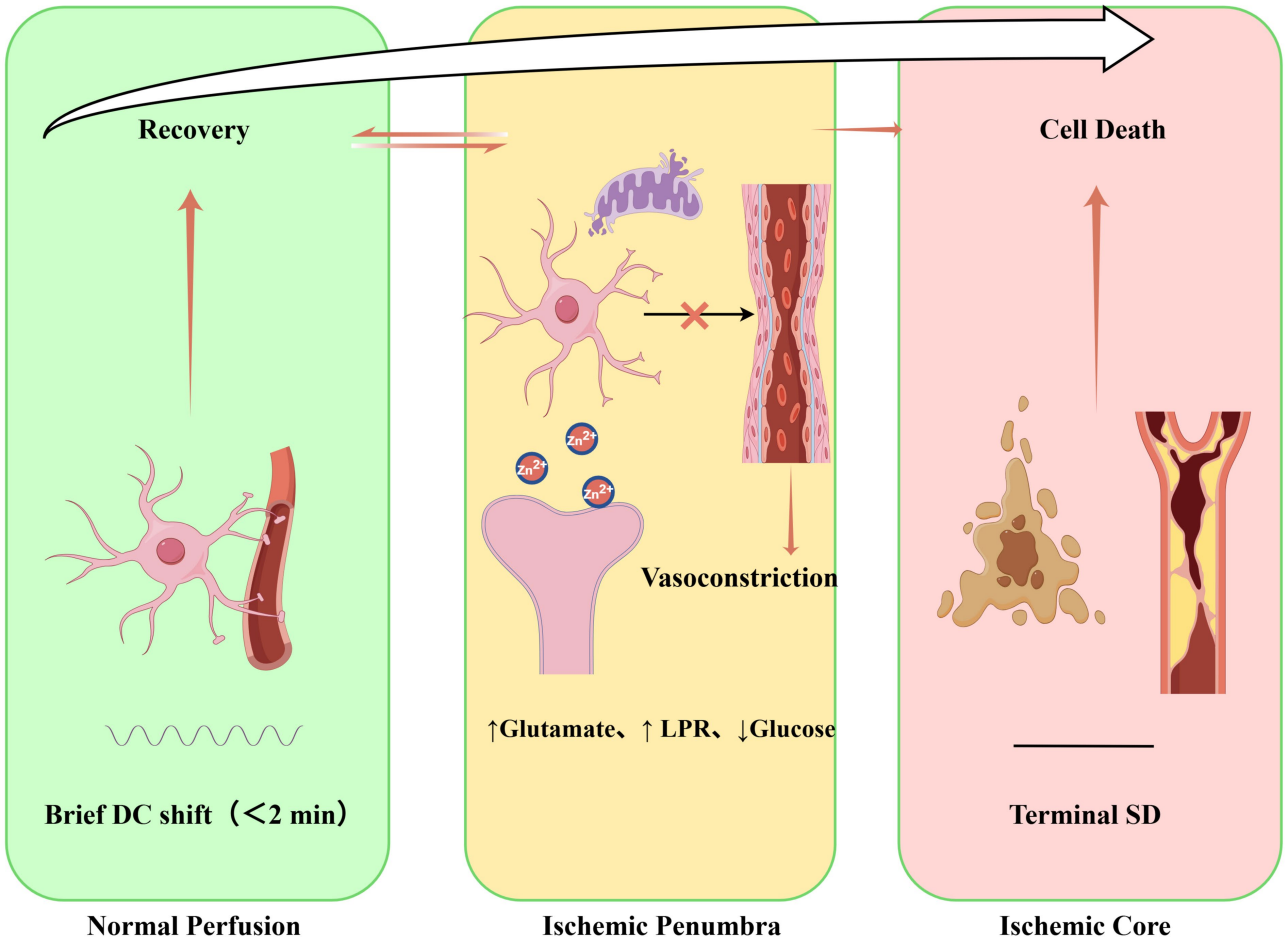
The pathophysiological continuum of spreading depolarization (SD): determinants and outcomes from benign to terminal SD.

While understanding SD genesis and propagation is crucial, the primary determinant of its clinical impact is the local tissue perfusion status at the time of the event, prompting an examination of SD effects under varying cerebral blood flow conditions (Figure 2).

### 2.2 Tissue perfusion dictates outcomes

The effect of an SD wave is wholly contingent upon the metabolic condition of the tissue it traverses (Table 1).

**Table 1:** Stratification of SD outcomes based on tissue perfusion.

Perfusion status (rCBF)	Electrophysiological signature	Metabolic & hemodynamic response	Clinical implications & tissue fate
Normal (>23 mL/100 g/min)	Brief negative DC shift (<2 min), followed by rapid repolarization and recovery of spontaneous activity.	Physiological neurovascular coupling: transient hyperemia meets metabolic demand. No significant ionic or metabolic disturbance.	Benign or protective: May contribute to ischemic preconditioning. No net injury.
Penumbra (15–23 mL/100 g/min)	Prolonged DC shift (>3- 5 min), slow/incomplete repolarization, prolonged suppression of activity.	Neurovascular uncoupling: Pathological vasoconstriction or inadequate hyperemia leads to spreading ischemia. Marked metabolic crisis (↑Glutamate, ↑LPR).	At-risk tissue: SD (often termed PID exacerbates supply)–demand mismatch promoting infarct expansion.
Ischemic core (<15 mL/100 g/min)	Terminal SD (TSD): Irreversible, massive DC shift with permanent electrical silence. No recovery of activity.	Perfusion is absent or minimal. Catastrophic failure of energy metabolism and ion homeostasis.	Cell death: Establishes the infarct core. SD waves terminate at its border.

In adequately perfused tissue, SD is transient and may even confer ischemic tolerance. In the energy-depleted penumbra, SD exacerbates the supply–demand mismatch, accelerating terminal depolarization and infarct growth—a process termed peri-infarct depolarization (PID; von Bornstädt et al., 2015). This continuum underscores SD’s dual role as both an indicator of tissue vulnerability and a facilitator of further damage.

As summarized in Table 1, the outcome of SD is critically dependent on tissue perfusion, underscoring that maintaining adequate cerebral perfusion pressure in neurocritical care is essential to mitigate its injurious potential. This schematic illustrates how the clinical significance of spreading depolarization (SD) is determined by the perfusion status of the brain tissue in which it occurs. In normally perfused tissue, SD is transient and benign. In the penumbra, neurovascular coupling failure leads to spreading ischemia and metabolic crisis, transforming SD into a deleterious event that actively promotes infarct expansion (i.e., peri-infarct depolarization, PID). In the ischemic core, SD represents a terminal manifestation of energy failure. This continuum underscores the importance of maintaining adequate cerebral perfusion to mitigate the injurious potential of SD and highlights the penumbra as a critical therapeutic window.

## 3 Surveillance methodologies

### 3.1 Invasive procedures

Subdural electrode strips for direct-current electrocorticography (DC-ECoG) represent the gold standard for clinical SD monitoring, typically placed during neurosurgical interventions (e.g., decompressive craniectomy, aneurysm clipping; Dreier et al., 2017; Hartings et al., 2017; Palopoli-Trojani et al., 2024).

DC-ECoG: Records the slow potential change (<0.05 Hz) characteristic of SD, manifested as a pronounced negative shift.

AC-ECoG: Reveals the suppression of faster neuronal activity (CSD) that follows the DC shift.

Multimodal monitoring integrates SD data with additional parameters, significantly enhancing clinical interpretation.

Cerebral Microdialysis: SD correlates with distinct metabolic crises, including surgers in extracellular glutamate, elevated lactate/pyruvate ratio, and significant glucose depletion (Rogers et al., 2017).

Tissue oxygenation (PbtO₂) and cerebral blood flow (CBF): In compromised tissue, SD can induce futher reductions in PbtO₂ and CBF, exemplifying spreading ischemia (Dreier et al., 2009; Hecht et al., 2025; Bosche et al., 2010).

### 3.2 Non-invasive innovations

The invasive nature of ECoG limits its widespread application. Scalp EEG has emerged as a viable non-invasive alternative. Advanced signal processing and machine learning techniques may detect ultra-slow potential shifts indicative of SD in full-band EEG, albeit with somewhat reduced sensitivity compared to ECoG (Drenckhahn et al., 2012; Bastany et al., 2016). This approach may facilitate the broader implementation of SD monitoring beyond specialized centers.

Recent scalp EEG innovations report SD detection sensitivity around 85% (Palopoli-Trojani et al., 2024; Chamanzar et al., 2023):

Algorithmic improvements: Machine learning classifiers (AUC = 0.91).

High-density arrays: 256-channel systems improve spatial localization.

Novel non-invasive biomarkers are also being investigated (Helbok et al., 2017):

Elevations of serum S100β following SD clusters (*r* = 0.79 with cluster frequency).

MRI diffusion tensor imaging: Anisotropy changes may predict SD pathways.

However, it is important to weigh these promising results against current limitations. Although studies like Drenckhahn et al. report correlation between scalp EEG and ECoG, the sensitivity of non-invasive methods remains lower than that of invasive monitoring, and they are less effective at detecting deep or subcortical SD events. Future efforts should integrate higher-density electrode arrays and optimized algorithms to overcome these shortcomings.

## 4 Clinical applications

### 4.1 Traumatic brain injury

Severe dysautonomia occurs in approximately 50% of observed severe traumatic brain injury patients (Hartings et al., 2011a; Chase, 2014). It manifests in two distinct temporal patterns: an early phase (peaking within 36 h) associated with ionic shifts and excitotoxicity, and a late phase (days 6–7) linked to evolving metabolic crises (Hartings et al., 2009). SD clusters and isoelectric SD (occurring in an electrically silent brain) are significant, independent predictors of adverse radiological and clinical outcomes, including mortality and severe disability (Hartings et al., 2011b; Abdelmalik et al., 2019).

### 4.2 Aneurysmal subarachnoid hemorrhage

SD is a fundamental mechanism in both early brain injury and delayed cerebral ischemia (DCI), which typically manifests 4–14 days post-ictus. Subarachnoid blood and its degradation products potently induce SD and impair neurovascular coupling (Turner, 2014; Dreier et al., 2022). The peak total depression duration per day (PTDDD), a measure of the cumulative burden of electrocorticographic suppression due to SD, has emerged as a potential biomarker for predicting DCI and reversible neurological deficits (Winkler et al., 2017; Sugimoto and Chung, 2020; Owen et al., 2022).

### 4.3 Malignant hemispheric stroke

SD is nearly ubiquitous in MHS, observed in virtually all monitored cases (Woitzik et al., 2013). Secondary injury, frequently observed as PIDs, spreads from the ischemic core toward the penumbra, leading to its gradual incorporation into the infarct. The correlation between SD burden and final infarct volume is robust, underscoring its role as a key contributor to secondary damage (von Bornstädt et al., 2015).

### 4.4 Cardiac arrest

There is increasing evidence that SD contributes to brain injury after to cardiac arrest. SD is prevalent among comatose patients following cardiac arrest and may exacerbate the advancement of anoxic damage. Therapeutic hypothermia, the established standard of care, seems to inhibit the incidence and propagation of spreading depolarization, indicating that a component of its neuroprotective mechanism may involve the modulation of this process (Han et al., 2022; Loonen et al., 2019; Aiba et al., 2016).

## 5 Therapeutic implications and prospective directions

The penumbral tissue, where SD propagates reversibly for a time, represents a crucial therapeutic window. The goal is to suppress SD or mitigate their deleterious effects to prevent infarct progression.

Pharmacological Targeting: NMDAR antagonists: Ketamine is the most extensively studied agent. Evidence from clinical and translational studies indicates a dose-dependent inhibition of SD, with dosages above approximately 1.15 mg/kg/h appearing more effective (Carlson et al., 2019; Sánchez-Porras et al., 2022; Reinhart et al., 2024). Memantine, another NMDAR antagonist, has shown efficacy in preclinical models (MacLean et al., 2023; Reinhart et al., 2021).

Other agents: Adrenergic receptor antagonists and gabapentin have demonstrated potential in modulating SD susceptibility in animal studies, offering alternative management avenues (Monai et al., 2019; Hoffmann et al., 2010; Monai et al., 2021).

Translating these pharmacological approaches into clinical practice requires careful consideration of context-specific cerebrovascular limitations (Lapchak and Zhang, 2017). When using NMDAR antagonists like ketamine, it is crucial to titrate the dose carefully while monitoring intracranial pressure and cerebral perfusion dynamics (De Sloovere et al., 2025). Novel therapeutic strategies may involve combination therapies that simultaneously inhibit SD propagation, enhance from collateral circulation, and modulate thrombo-inflammatory processes (Chen et al., 2017; Vitale et al., 2023; Yoshiura et al., 2025; Fischer et al., 2023).

Non-Pharmacological Strategies: A fundamental approach to modulating SD is the optimization of systemic physiological parameters. Avoiding hypotension, hypoglycemia, hyperthermia, and severe intracranial hypertension can reduce the brain’s susceptibility to SD (Hartings et al., 2009; Nash et al., 2023).

**Figure 3:**
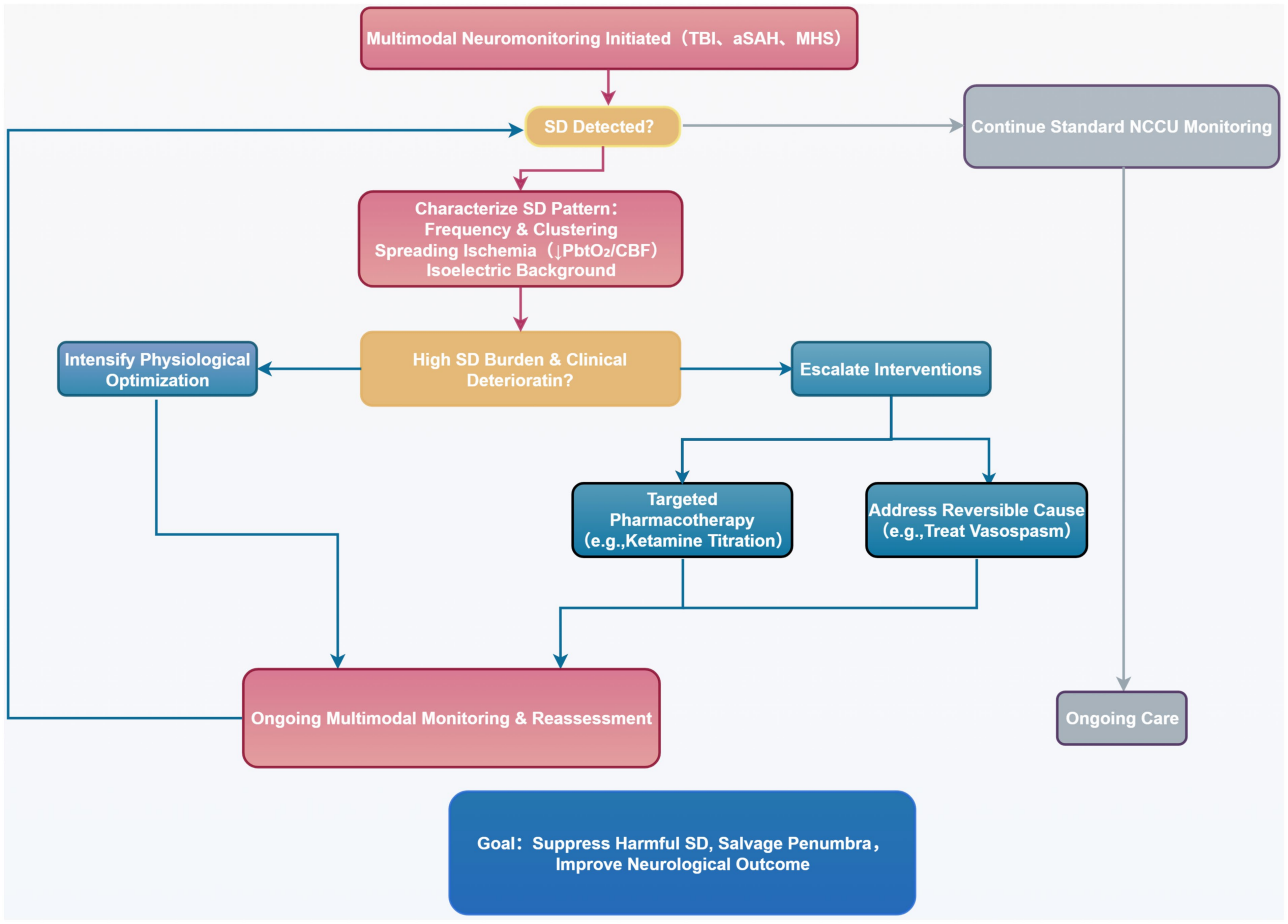
Proposed decision pathway for precision management guided by spreading depolarization monitoring in neurocritical care.

Incorporating SD monitoring into multimodal neuromonitoring protocols represents a transformative advance in neurocritical care (Hartings et al., 2020; Veldeman et al., 2021; Gouvêa Bogossian et al., 2025; Ayata, 2018; Yang, 2020). Real-time detection of SD clusters allows for the identification of patients at imminent risk of secondary neurological deterioration, enabling proactive neuroprotective interventions. Within precision neurocritical care frameworks, a high SD burden may trigger clinical escalation, such as intensified hemodynamic optimization, consideration of early decompressive craniectomy, or initiation of targeted anti-depolarization therapy. Figure 3 illustrates an example of a clinical decision pathway based on SD monitoring. However, translating SD suppression into improved clinical outcomes remains the paramount challenge. Future efforts should focus on large-scale, randomized controlled trials targeting patients with significant SD burden, utilizing multimodal monitoring to guide personalized therapy (van Hameren et al., 2024).

## 6 Conclusion

SD represents a central electrophysiological signature of cerebral metabolic failure cascades in neurocritical care and is increasingly recognized as vital for evidence-based management of cerebrovascular pathology (Dreier et al., 2017; Hartings, 2017; Alkhachroum et al., 2022). This review has highlighted collaborative progress in defining its nomenclature, detecting it non-invasively, interpreting it within a multimodal framework, and strategically targeting it with emerging, dose-dependent therapeutics. Incorporating real-time SD dynamics into therapeutic decision-making holds significant promise for advancing precision medicine in secondary brain injury. To realize this potential into real benefits, future work must prioritize the validation of SD-guided intervention protocols through large-scale, randomized trials targeting high-risk phenotypes, concurrently advancing robust, real-time analytical tools for clinical deployment.
